# The belief that politics drive scientific research & its impact on COVID-19 risk assessment

**DOI:** 10.1371/journal.pone.0249937

**Published:** 2021-04-21

**Authors:** Danielle M. McLaughlin, Jack Mewhirter, Rebecca Sanders

**Affiliations:** Department of Political Science, University of Cincinnati, Cincinnati, OH, United States of America; University of Haifa, ISRAEL

## Abstract

We use survey data collected from 12,037 US respondents to examine the extent to which the American public believes that political motives drive the manner in which scientific research is conducted and assess the impact that such beliefs have on COVID-19 risk assessments. We find that this is a commonly held belief and that it is negatively associated with risk assessments. Public distrust in scientists could complicate efforts to combat COVID-19, given that risk assessments are strongly associated with one’s propensity to adopt preventative health measures.

## Introduction

More than a year after the Centers for Disease Control confirmed the first US COVID-19 case, the novel coronavirus continues to exact a devastating toll. Scientists and public health experts have repeatedly informed the public of the dangers posed by COVID-19 and advocated for the adoption of evidence-based practices (e.g., social distancing, facemasks, etc.) designed to reduce transmission rates. Despite these efforts, many Americans assert that the risks associated with COVID-19 have been largely exaggerated––a sentiment associated with resistance to adopting preventative health measures [1–6]. In this article, we examine how one specific factor impacts COVID-19 risk assessments: the belief that political motives drive the manner in which scientists conduct their research. Using survey data collected from a nationally representative sample of US residents, we find that i) a large portion of the population believes that politics drive the manner in which scientific studies are conducted, and ii) holding such a belief has a strong, negative association with COVID-19 risk assessments.

### The belief that scientific research is politically motivated: Origins and impacts

A fundamental role of scientific research is to provide objective evidence that can be used to inform policy debates [7]. Despite this standard, scholarship shows that the American public is increasingly concerned about the objectivity of scientific research, reflecting and reinforcing growing anti-science sentiment [8, 9]. Public perceptions that scientific research is politically motivated emanates from several sources. Most notably, politicians and media can fuel these perceptions by i) presenting partial or misleading accounts of scientific methods and findings to legitimize political agendas and ii) discrediting scientists with accusations of collusion with political interests (e.g., “Big Pharma”) [7, 8, 10–19]. For instance, President Trump claimed that the Food and Drug Administration was intentionally delaying COVID-19 drug trials to hurt his reelection: “The deep state, or whoever, over at the FDA is making it very difficult for drug companies to get people in order to test the vaccines and therapeutics” [20]. Similarly, during the pandemic, a number of news personalities consistently questioned the character of the “so called experts” who developed the Imperial College’s influential COVID-19 mortality rate and healthcare demand models [21].

Sowing doubt over the impartiality and validity of scientific research and/or disparaging scientists’ intentions and credibility may induce public skepticism about purported COVID-19 risks and science-based policy responses [10, 11, 17–19, 22–24]. Accusations that science is “biased” or “rigged” may trigger an emotive audience response, stimulating a status quo bias whereby individuals repudiate risk and reject the adoption of new preventative measures advocated by scientists [22, 24–29]. This has significant implications because those holding lower risk assessments are more likely to eschew measures (e.g., facemasks, social distancing, vaccines) designed to safeguard communities [1–6]. Our empirical approach, described below, uses survey data to discern i) the extent to which the American public believes that politics drive scientific research, and ii) the impact that such beliefs have on COVID-19 risk assessments.

## Materials and methods

### Ethics approval and consent

The research team obtained informed consent from all subjects. At the beginning of the survey, respondents were presented with an information sheet detailing the terms of the study. Respondents were informed that “BY TAKING PART IN THESE ACTIVITIES YOU INDICATE YOUR CONSENT FOR YOUR ANSWERS TO BE USED IN THIS RESEARCH STUDY”. Note that the researchers obtained a waiver documentation (signature) of consent from the University of Cincinnati Institutional Review Board (FWA #: 000003152).

To evaluate our contention, this study utilizes survey data collected from American adults. Respondents to our web-based survey were recruited via the internet panel provider, Qualtrics, which manages participant compensation and quality control [30]. Qualtrics distributed the survey between August and September of 2020. Quotas based on race, gender, age, and census statistical division, matched to 2018 census estimates, were included to ensure a demographically and geographically representative sample of the United States adult population. Each respondent was compensated for their participation in accordance with their agreement with Qualtrics. Respondents were screened for quality by Qualtrics (e.g., for speed and random response). The data collection yielded a total of 13,373 responses. Of those, 1,336 responses were deemed low quality, resulting in a final sample size of 12,037.

### Dependent variable

The dependent variable—*Risk-Index*—assesses the level of COVID-19 related risk one perceives related to a number of daily activities. Respondents were asked to report on a 0–10 scale how safe—where 0 indicates “very unsafe” and 10 indicates “very safe”—they would feel doing the following activities: “going to the grocery store”, “going to work with others”, “eating in a restaurant”, “sending a child to school or daycare”, “attending a religious ceremony”, “attending a large public event”, and “voting in person”. To generate the index, we reverse coded each component (10-reported value) and took the mean value. A Cronbach’s alpha test was conducted to assess the internal consistency of the components. The alpha coefficient (0.9339) reveals high reliability, thus justifying use of an index.

### Independent variable

We use responses to the following statement to construct the variable *Science Trust—Apolitical Motivations*: “A lot of research conducted by scientists is driven by their political motives”. Values range from 0–10, where 0 indicates complete disagreement, and 10 indicates complete agreement. These values were then reverse coded (10-reported value). This measure was adapted from a question in the General Social Survey [31].

### Control variables

We include a number of control variables that capture i) alternative forms of trust in the scientific community, ii) trust in politicians, media, and how each uses/communicates science, iii) how an individual and people in their network have been physically, financially, and mentally impacted by the pandemic, iv) dogmatism, v) health and risk-factors, vi) knowledge of science, vii) beliefs regarding the role of individuals in creating and addressing/mitigating the pandemic, viii) various individual attributes (race, age, education, gender, income, and political affiliation). Summary statistics for all variables are included in the S1 Table in S1 File. All replication materials can be accessed through the Harvard Dataverse (https://doi.org/10.7910/DVN/U6Q5FV).

#### Different forms of trust in the scientific community

We include two variables, *Science Trust—Betterment* and *Science Trust—Community*, that capture a respondent’s general level of trust in the scientific community. Including these variables in our model allows us to disaggregate the impact of one specific form of trust—trust that political motivations do not impact the manner in which research is conducted—from the broader concept of “trust in science”. To construct these variables, participants were asked to respond to the following statements: “Most scientists want to work on things that will make life better for the average person” and “I have a great deal of confidence in the people running the scientific community”. Values range from 0–10 where 0 indicates complete disagreement and 10 indicates complete agreement.

#### Trust in the government, media, and their respective use of scientific research

We anticipate that distrust in elites (e.g., government, media, etc.) and the means by which elites use and communicate scientific findings could spill over into sentiments regarding scientists’ research motives. Moreover, individuals who distrust the way that elites convey scientific information to the public may actively avoid and/or dismiss information transfers from such actors—in this case, COVID-19 related information—leading to lower levels of COVID-19 related knowledge [32–36], and subsequently, lower risk assessments.

We asked respondents how often they can trust the national government, state government, local government, and the news media “to do what is right”. Responses range from 0–10 where 0 indicates “never” and 10 indicates “always”. *Government Trust* takes the mean value of the first three components, whereas *Media Trust* is the reported value of the latter. We also capture trust in how media and politicians use scientific information. Participants were asked to identify the extent to which they agree or disagree with the following statements: “The news media often skews and misrepresents scientific findings to promote their own interests”; “Politicians often skew and misrepresent scientific findings to promote their own interests”. Values range from 0–10 where 0 indicates complete disagreement and 10 indicates complete agreement. These values were reverse coded (10-reported value) to create the variables *Use of Science—Government* and *Use of Science—Media*.

#### Pandemic impact: Personal & network

We include a number of variables that account for the physical, financial, and mental toll that the pandemic and resulting government response has taken on individuals and those they consider close to them. Prior research demonstrates an intimate connection between personal experience and risk processing [1, 37]. When the virus hits close to home—personally, financially, or mentally—risk perceptions may be heightened by activating the affective experience [1, 37]. Simultaneously, such experiences may also promote openness to scientific learning [38, 39], whereby people may be more inclined to learn from and trust reliable scientific sources.

*Personal Impact—Infected* indicates whether an individual has tested positive for COVID-19 (0 = No; 1 = Yes), whereas *Network Impact—Infected* indicates whether someone close to them tested positive (0 = No; 1 = Yes). *Personal Impact—Finances* and *Network Impact—Finances* report how an individual and those they consider close to them were financially impacted by the virus. Values range from 0–10 where 0 indicates a major negative impact, and 10 indicates a major positive impact. *Personal Impact—Mental* and *Network Impact—Mental* mirror the aforementioned measures but relate to mental instead of financial health.

#### Risk factors

We also include variables that indicate whether an individual has a number of medical conditions that place them in a high-risk category for COVID-19 related mortality [40]. We anticipate that those with such conditions will hold more positive views about the scientific research community and will have heightened risk assessments. We include variables that identify whether an individual has one of the following conditions (= 1) or not (= 0): asthma; lung disease; diabetes; immune disorder; pregnancy; obesity; heart problems; liver or kidney problems.

#### Scientific knowledge

*Scientific Literacy* gauges one’s level of scientific knowledge. Respondents were asked a number of science-based trivia questions: the final measure reports the proportion of correct responses (0–1). Participants were asked to respond, “True or False” to the following statements: “all radioactivity is man-made”; “the sun revolves around the earth”; “the continents on which we live have been moving their locations for millions of years and will continue to move in the future”; “the center of the earth is very hot”; “antibiotics kill viruses and bacteria”; “vaccines help develop immunity to disease”. These questions were largely taken from the General Social Survey [31]. Those with higher scientific literacy are likely more attuned to the objective nature of science and more trusting of its sources [41], thus heeding calls to behave cautiously during the pandemic.

#### Blame attribution

*Individuals to Blame* and *Individual Responsibility* capture the extent to which an individual feels like the public (and not another entity: e.g., government, researchers, etc.) are to blame for the pandemic and should be responsible for solving it. To create the measures, participants were asked to respond to the following statements: “Most coronavirus cases could have been prevented if people were more cautious”; “It is the responsibility of every individual, not the government, to protect themselves during times of crisis”. Values range from 0–10 where 0 indicates complete disagreement and 10 indicates complete agreement. We anticipate that attributions of blame and responsibility for the state of affairs surrounding a certain event may impact attitudes toward decision-makers and policy issues as well as individual behavior [42, 43].

#### Dogmatism

Next, a *Dogmatism-Index* was constructed by taking the mean value assigned to each of the following statements: “There are two kinds of people in this world: those who are for the truth and those who are against it”; “A group that tolerates too many differences of opinion among its members cannot exist for long”; “To compromise with our political opponents is dangerous because it usually leads to the betrayal of our own side”; “Of all the different philosophies that exist in the world there is probably only one that is correct”; “In the long run the best way to live is to pick friends and associates whose tastes and beliefs are the same as one’s own”. The index mirrors that of Davis and Silver [39] who argue that dogmatic people are inherently inflexible and more likely to “take an either-or-approach” in complex situations such as a global health pandemic. We expect such closed belief systems will impact i) one’s propensity to trust the impartiality of scientific research and ii) one’s information processing regarding COVID risks.

#### Personal attributes

Finally, several variables are included that historically influence one’s tendency to trust elites and evaluate risks associated with public policies [39, 44, 45]. *College Graduate* indicates whether the individual completed a four-year degree (= 1) or not (= 0). *Age* indicates a person’s age in years. *Race* indicates whether a respondent is white (= 0), Hispanic (= 1), Black (= 2), or another race (= 3). *Political Party* indicates whether a respondent is a Democrat (= 0), Independent (= 1), Libertarian (= 2), Republican (= 4), or something else (= 5). *Trump* indicates whether a respondent approves of the job Donald Trump is doing as president (= 1) or not (= 0).

#### Statistical approach

We estimate a multilevel regression model with random intercepts at the state level to examine how increased beliefs that science is apolitical affect the level of risk one perceives performing daily tasks. Inclusion of random intercepts accounts for unmodeled, state-specific factors (infection rates, culture, government response, etc.) that could impact estimates [46]. We include a quadratic term of our independent variable as the effect proved to be non-linear. Inclusion of the quadratic term led to improved goodness of fit (AIC = 47945.37, BIC = 48231.27) relative to a model without the term (AIC = 48013.00, BIC = 48291.58). The results from the alternate model are presented in the S2 Table in S1 File, column 2.

## Results

**Fig 1:**
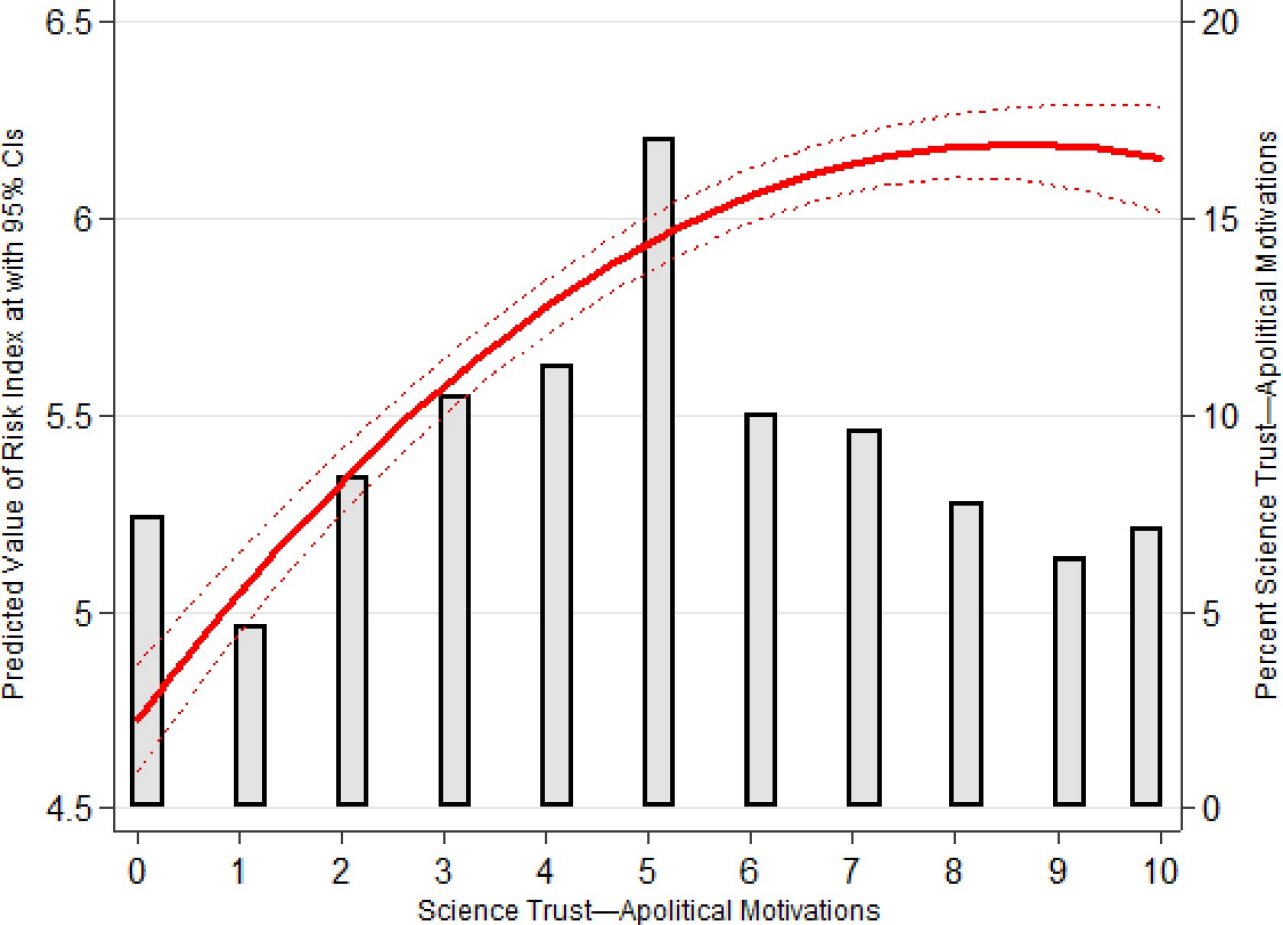
Predicted values of *Risk Index* across values of *Science Trust—Apolitical Motivations* with embedded histogram.

Fig 1 presents a histogram detailing the distribution of the independent variable, as well as the predicted values of *Risk-Index* across all values of the independent variable. Full regression results are presented in Table 1. As shown in Fig 1, respondents, on average, are not confident that scientific research is apolitical: 31.21% believe that science is politically motivated (values 0–3), 20.81% believe that it is not (values 7–10), and 27.19% are fairly ambivalent (values 4–6).

**Table 1:** Regression results for primary multilevel regression model and alternate model excluding the *Science Trust—Apolitical*^*2*^.

	Primary Model
*Science Trust—Apolitical*	0.337***
	(0.025)
*Science Trust—Apolitical*^*2*^	-0.020***
	(0.002)
*Science Trust—Betterment*	0.039***
	(0.011)
*Science Trust—Community*	-0.004
	(0.011)
*Government Trust Index*	-0.143***
	(0.012)
*Media Trust*	0.033***
	(0.010)
*Use of Science—Government*	0.035***
	(0.010)
*Use of Science—Media*	0.087***
	(0.010)
*Personal Impact—Infected*	-1.044***
	(0.119)
*Network Impact—Infected*	0.178***
	(0.050)
*Personal Impact—Finances*	-0.056***
	(0.012)
*Personal Impact—Mental*	-0.061***
	(0.013)
*Network Impact—Finances*	-0.007
	(0.012)
*Network Impact—Mental*	-0.044***
	(0.013)
*Scientific Literacy*	-0.210*
	(0.086)
*Individuals to Blame*	0.243***
	(0.009)
*Individual Responsibility*	-0.117***
	(0.008)
*Dogmatism Index*	-0.108***
	(0.012)
*Race*: *Reference = White*	
*Black*	0.089
	(0.067)
*Hispanic*	0.136*
	(0.057)
*Other*	0.518***
	(0.069)
*College Graduate*	-0.126**
	(0.041)
*Age*	0.010***
	(0.001)
*Political Party*: *Reference = Democrat*	
*Independent*	-0.108*
	(0.051)
*Libertarian*	-0.590***
	(0.154)
*Other*	0.030
	(0.101)
*Republican*	-0.308***
	(0.061)
*Trump Approval*	-0.795***
	(0.056)
*Risk—Pregnant*	-0.288*
	(0.145)
*Risk—Asthma*	0.114
	(0.061)
*Risk—Lung Disease*	0.217
	(0.131)
*Risk—Diabetes*	0.049
	(0.063)
*Risk—Immune Disorder*	0.496***
	(0.083)
*Risk—Obesity*	0.015
	(0.063)
*Risk—Heart Problem*	0.259**
	(0.082)
*Risk—Liver or Kidney Problem*	-0.132
	(0.134)
*Constant*	5.090***
	(0.163)
*RE Variance*: *State*	0.131***
	(0.030)
*LR Test*: *χ*^*2*^	19.95***
*AIC*	47945.37
*BIC*	48231.27
Observations	11,281
Number of groups	51

Standard errors in parentheses: *** p<0.001,

** p<0.01,

* p<0.05.

Results show that greater confidence that science is apolitical is associated with higher risk assessments, though this effect diminishes at higher values of the independent variable (*Science Trust—Apolitical Motivations*: b = .34; z = 13.38; *Science Trust—Apolitical Motivations*^*2*^ b = -.02; z = -8.36). The impact is striking: those who completely disagree that scientific research is apolitical have an expected *Risk-Index* score of 4.74, compared to 6.16 for those who completely agree, a 23.06% decrease.

The results also demonstrate that *Science Trust—Betterment* is positively associated with risk assessments, though the substantive impact is considerably smaller: those who completely disagree that most scientists work on projects that positively impact the average person have an expected *Risk-Index* score 6.61% lower than those who completely agree, holding all else constant. The effect of *Science Trust—Community* does not attain statistical significance.

While one may be concerned that the variation in effect between *Science Trust—Apolitical Motivations* and the alternate *“Science Trust” variables* is attributable to the terms being highly correlated, this is not the case: *Science Trust—Apolitical Motivations* is only moderately correlated with *Science Trust—Betterment* (r = .29) and *Science Trust—Community* (r = .22). As such, our independent variable represents an independent construct: one that is strongly associated with risk assessments. *Science Trust—Betterment* and *Science Trust—Community* are highly correlated (r = .61). In the S2 Table in S1 File, column 3, we present regression estimates with *Science Trust—Community* dropped from the model. As shown, the coefficient estimates of the remaining *Science Trust* variables are entirely consistent with those in Table 1, confirming that *Science Trust—Apolitical* has a larger substantive impact on risk assessments. While speculative, we contend that this disparity may be due to highly visible and politically polarizing allegations by media and politicians that science has been politicized during the pandemic.

We conduct a number of robustness checks to assess the robustness of our findings. First, we calculate the variance inflation factor (S3 Table in S1 File) to test for the presence and impact of multicollinearity. Second, we estimate 10 alternate regression models to assess whether our results are dependent on variable inclusion (S4 Table in S1 File). As shown in the S1 File, results are robust to all specifications and multicollinearity does not meaningfully impact estimates.

## Discussion

Scientific norms extol that “scientific outcomes should not be predetermined by political perspectives”, as such manipulation is antithetical to the ideals of science [7]. However, calculated efforts to marginalize science have greatly impacted public opinion and policy on topics such as climate change, vaccinations, and stem cell research [7, 12, 13, 47, 48]. In line with these trends, our findings suggest that scientific consensus on the public health threat posed by COVID-19 has not persuaded all Americans. While now household names such as Doctors Fauci and Birx implore people to take extensive precautions to avoid contracting and spreading disease, their advice is discounted by those who believe science is driven by political motives. People who think that scientific research and recommendations are politically motivated are less likely to perceive risk and adopt preventative health measures and are consequently more likely to become victims and vectors of the virus.

Unlike ostensibly stable determinants of risk perception such as cultural, social, and religious characteristics [2, 49], perceptions related to the veracity of science *may* be more malleable. To improve compliance with their advice, stakeholders at the forefront of the COVID-19 crisis must convincingly communicate that public health recommendations have not been corrupted by political incentives. However, research suggests that scientists tend toward a deficit model when communicating with the public, believing that closing the public’s knowledge gap will improve support for new science policy [8]. Our results show that this is insufficient, and that scientists and public health leaders must strategize to directly counteract politicization accusations [1, 18, 50]. Counteraction measures such as warning people that they may be exposed to inaccurate information that goes against the scientific consensus and correcting such information when it is disseminated may temper the effects of perceptions of politicization [22, 48].

The belief that scientific research is politically motivated drastically reduces the public’s COVID-19 risk assessments. When it comes to public health, public trust in and cooperation with scientific advice is critical. As suffering wrought by COVID-19 intensifies, addressing perceptions of science politicization is more urgent than ever. While our results are compelling, limitations remain. First, our data is restricted to the United States and therefore, we cannot examine potential international differences. Such comparisons would be especially insightful given the somewhat unique historical moment the United States is embroiled in, at the intersection of a pandemic, civil unrest, and a polarizing political leader. Second, while our results are strongly suggestive and robust to different model specifications, our reliance on cross-sectional observational data precludes us from making causal claims due to the potential for omitted variable bias and simultaneity bias. Although we theorize on the origins of beliefs that science is politically motivated, future work should seek to more explicitly examine the causes underlying politicization perceptions.

## Supporting information

S1 File (DOCX)
