# Supplementary material for: The belief that politics drive scientific research & its impact on COVID-19 risk assessment
Source: PLoS One. 2021 Apr 21;16(4):e0249937. doi: 10.1371/journal.pone.0249937 (PMC8059820; doi:10.1371/journal.pone.0249937)
Supplement: S1 File — (DOCX) [file pone.0249937.s001.docx]

**S1 Table. Summary Statistics for all Variables Used in Analyses**

|  | **N** | **Mean** | **SD** | | **Min** | **Max** |
| --- | --- | --- | --- | --- | --- | --- |
| *Risk-Index* | 12,008 | 5.781 | 2.608 | | 0 | 10 |
| *Science Trust—Apolitical* | 11,995 | 4.982 | 2.794 | | 0 | 10 |
| *Science Trust—Betterment* | 11,987 | 6.945 | 2.232 | | 0 | 10 |
| *Science Trust—Community* | 11,989 | 6.244 | 2.470 | | 0 | 10 |
| *Government Trust Index* | 12,008 | 5.355 | 2.145 | | 0 | 10 |
| *Media Trust* | 11,996 | 4.610 | 2.911 | | 0 | 10 |
| *Use of Science—Government* | 11,989 | 2.575 | 2.325 | | 0 | 10 |
| *Use of Science—Media* | 11,992 | 3.450 | 2.835 | | 0 | 10 |
| *Personal Impact—Infected* | 12,023 | .0323 | .176 | | 0 | 1 |
| *Network Impact—Infected* | 12,020 | .1970 | .397 | | 0 | 1 |
| *Personal Impact—Finances* | 11,990 | 5.036 | 2.531 | | 0 | 10 |
| *Personal Impact—Mental* | 11,971 | 4.815 | 2.506 | | 0 | 10 |
| *Network Impact—Finances* | 11,963 | 4.98 | 2.496 | | 0 | 10 |
| *Network Impact—Mental* | 11,958 | 4.791 | 2.399 | | 0 | 10 |
| *Scientific Literacy* | 12,032 | .6786 | .263 | | 0 | 1 |
| *Individuals to Blame* | 12,002 | 7.227 | 2.424 | | 0 | 10 |
| *Individual Responsibility* | 11,997 | 6.632 | 2.643 | | 0 | 10 |
| *Dogmatism Index* | 12,007 | 4.956 | 2.046 | | 0 | 10 |
| *College Graduate* | 12,030 | .4789 | .499 | | 0 | 1 |
| *Age* | 12,025 | 47.002 | 17.868 | | 18 | 98 |
| *Trump Approval* | 12,017 | .387 | .487 | | 0 | 1 |
| *Risk—Pregnant* | 11,522 | .0184 | .134 | | 0 | 1 |
| *Risk—Asthma* | 11,522 | .114 | .318 | | 0 | 1 |
| *Risk—Lung Disease* | 11,522 | .022 | .148 | | 0 | 1 |
| *Risk—Diabetes* | 11,522 | .112 | .316 | | 0 | 1 |
| *Risk—Immune Disorder* | 11,522 | .0567 | .231 | | 0 | 1 |
| *Risk—Obesity* | 11,522 | .109 | .312 | | 0 | 1 |
| *Risk—Heart Problem* | 11,522 | .062 | .242 | | 0 | 1 |
| *Risk—Liver or Kidney Problems* | 11,522 | .021 | .146 | | 0 | 1 |
|  | | | | | | |
|  | **N** | | | **%** | | |
| *Race—White* | 7,247 | | | 60.21 | | |
| *Race—Black* | 1,478 | | | 12.28 | | |
| *Race—Hispanic* | 2,119 | | | 17.61 | | |
| *Race—Other* | 1,192 | | | 9.90 | | |
| *Political Affiliation—Democrat* | 4,471 | | | 37.18 | | |
| *Political Affiliation—Independent* | 3,349 | | | 27.85 | | |
| *Political Affiliation—Libertarian* | 207 | | | 1.72 | | |
| *Political Affiliation—Other* | 554 | | | 4.61 | | |
| *Political Affiliation—Republican* | 3,444 | | | 28.64 | | |

**S2 Table: Regression Results for Primary Multilevel Regression Model and Alternate Model Excluding the *Science Trust—Apolitical^2^***

|  | Primary Model | Alternate Model  No Quadratic Term | Alternate Model  Drop *ST-Community* |
| --- | --- | --- | --- |
|  |  |  |  |
| *Science Trust—Apolitical* | 0.337*** | 0.142*** | 0.337*** |
|  | (0.025) | (0.009) | (0.025) |
| *Science Trust—Apolitical^2^* | -0.020*** |  | -0.020*** |
|  | (0.002) |  | (0.002) |
| *Science Trust—Betterment* | 0.039*** | 0.031** | 0.037*** |
|  | (0.011) | (0.011) | (0.010) |
| *Science Trust—Community* | -0.004 | -0.008 |  |
|  | (0.011) | (0.011) |  |
| *Government Trust Index* | -0.143*** | -0.137*** | -0.143*** |
|  | (0.012) | (0.012) | (0.011) |
| *Media Trust* | 0.033*** | 0.030** | 0.032*** |
|  | (0.010) | (0.010) | (0.010) |
| *Use of Science—Government* | 0.035*** | 0.050*** | 0.035*** |
|  | (0.010) | (0.010) | (0.010) |
| *Use of Science—Media* | 0.087*** | 0.082*** | 0.087*** |
|  | (0.010) | (0.010) | (0.010) |
| *Personal Impact—Infected* | -1.044*** | -1.083*** | -1.044*** |
|  | (0.119) | (0.119) | (0.119) |
| *Network Impact—Infected* | 0.178*** | 0.182*** | 0.179*** |
|  | (0.050) | (0.050) | (0.050) |
| *Personal Impact—Finances* | -0.056*** | -0.057*** | -0.057*** |
|  | (0.012) | (0.012) | (0.012) |
| *Personal Impact—Mental* | -0.061*** | -0.061*** | -0.061*** |
|  | (0.013) | (0.013) | (0.013) |
| *Network Impact—Finances* | -0.007 | -0.007 | -0.007 |
|  | (0.012) | (0.012) | (0.012) |
| *Network Impact—Mental* | -0.044*** | -0.044** | -0.045*** |
|  | (0.013) | (0.013) | (0.013) |
| *Scientific Literacy* | -0.210* | -0.227** | -0.213* |
|  | (0.086) | (0.086) | (0.086) |
| *Individuals to Blame* | 0.243*** | 0.243*** | 0.242*** |
|  | (0.009) | (0.009) | (0.009) |
| *Individual Responsibility* | -0.117*** | -0.118*** | -0.117*** |
|  | (0.008) | (0.008) | (0.008) |
| *Dogmatism Index* | -0.108*** | -0.113*** | -0.108*** |
|  | (0.012) | (0.012) | (0.012) |
| *Race: Reference=White* |  |  |  |
| *Black* | 0.089 | 0.085 | 0.092 |
|  | (0.067) | (0.067) | (0.067) |
| *Hispanic* | 0.136* | 0.137* | 0.135* |
|  | (0.057) | (0.057) | (0.057) |
| *Other* | 0.518*** | 0.539*** | 0.519*** |
|  | (0.069) | (0.069) | (0.069) |
| *College Graduate* | -0.126** | -0.130** | -0.126** |
|  | (0.041) | (0.041) | (0.041) |
| *Age* | 0.010*** | 0.010*** | 0.010*** |
|  | (0.001) | (0.001) | (0.001) |
| *Political Party: Reference=Democrat* |  |  |  |
| *Independent* | -0.108* | -0.094 | -0.108* |
|  | (0.051) | (0.051) | (0.050) |
| *Libertarian* | -0.590*** | -0.595*** | -0.589*** |
|  | (0.154) | (0.154) | (0.154) |
| *Other* | 0.030 | 0.031 | 0.022 |
|  | (0.101) | (0.101) | (0.101) |
| *Republican* | -0.308*** | -0.302*** | -0.307*** |
|  | (0.061) | (0.061) | (0.061) |
| *Trump Approval* | -0.795*** | -0.813*** | -0.793*** |
|  | (0.056) | (0.056) | (0.055) |
| *Risk—Pregnant* | -0.288* | -0.318* | -0.287* |
|  | (0.145) | (0.146) | (0.145) |
| *Risk—Asthma* | 0.114 | 0.102 | 0.114 |
|  | (0.061) | (0.061) | (0.061) |
| *Risk—Lung Disease* | 0.217 | 0.214 | 0.216 |
|  | (0.131) | (0.132) | (0.131) |
| *Risk—Diabetes* | 0.049 | 0.033 | 0.050 |
|  | (0.063) | (0.063) | (0.063) |
| *Risk—Immune Disorder* | 0.496*** | 0.481*** | 0.496*** |
|  | (0.083) | (0.084) | (0.083) |
| *Risk—Obesity* | 0.015 | 0.019 | 0.018 |
|  | (0.063) | (0.063) | (0.063) |
| *Risk—Heart Problem* | 0.259** | 0.264** | 0.259** |
|  | (0.082) | (0.082) | (0.082) |
| *Risk—Liver or Kidney Problem* | -0.132 | -0.155 | -0.133 |
|  | (0.134) | (0.134) | (0.134) |
| *Constant* | 5.090*** | 5.529*** | 5.097*** |
|  | (0.163) | (0.155) | (0.163) |
| *RE Variance: State* | 0.131*** | 0.129*** | 0.131*** |
|  | (0.030) | (0.030) | (0.030) |
| *LR Test: χ^2^* | 19.95*** | 18.89*** | 19.74*** |
| *AIC* | 47945.37 | 48013.00 | 47970.87 |
| *BIC* | 48231.27 | 48291.58 | 48249.46 |
| Observations | 11,281 | 11,281 | 11,287 |
| Number of groups | 51 | 51 | 51 |

*Standard errors in parentheses: *** p<0.001, ** p<0.01, * p<0.05*

**S3 Table. Variance Inflation Factor Test for Primary Multilevel Regression Model and Alternate Model Excluding the *Science Trust—Apolitical^2^***

|  | VIF: Primary Model | VIF: Alternate Model |
| --- | --- | --- |
| *Science Trust—Apolitical* | 13.81 | 1.91 |
| *Science Trust—Apolitical^2^* | 13.13 |  |
| *Science Trust—Betterment* | 1.77 | 1.76 |
| *Science Trust—Community* | 2.01 | 2.01 |
| *Government Trust Index* | 1.69 | 1.68 |
| *Media Trust* | 2.23 | 2.22 |
| *Use of Science—Government* | 1.40 | 1.35 |
| *Use of Science—Media* | 2.03 | 2.03 |
| *Personal Impact—Infected* | 1.17 | 1.17 |
| *Network Impact—Infected* | 1.10 | 1.10 |
| *Personal Impact—Finances* | 2.35 | 2.35 |
| *Personal Impact—Mental* | 2.71 | 2.71 |
| *Network Impact—Finances* | 2.35 | 2.35 |
| *Network Impact—Mental* | 2.81 | 2.81 |
| *Scientific Literacy* | 1.36 | 1.36 |
| *Individuals to Blame* | 1.21 | 1.21 |
| *Individual Responsibility* | 1.34 | 1.34 |
| *Dogmatism Index* | 1.73 | 1.72 |
| *Race: Reference=White* |  |  |
| *Black* | 1.29 | 1.29 |
| *Hispanic* | 1.23 | 1.23 |
| *Other* | 1.12 | 1.12 |
| *College Graduate* | 1.15 | 1.15 |
| *Age* | 1.45 | 1.45 |
| *Political Party: Independent* | 1.41 | 1.41 |
| *Political Party: Libertarian* | 1.06 | 1.06 |
| *Political Party: Other* | 1.13 | 1.13 |
| *Political Party: Republican* | 2.12 | 2.12 |
| *Trump Approval* | 2.03 | 2.02 |
| *Risk—Pregnant* | 1.04 | 1.04 |
| *Risk—Asthma* | 1.05 | 1.05 |
| *Risk—Lung Disease* | 1.04 | 1.04 |
| *Risk—Diabetes* | 1.11 | 1.10 |
| *Risk—Immune Disorder* | 1.04 | 1.04 |
| *Risk—Obesity* | 1.06 | 1.06 |
| *Risk—Heart Problem* | 1.08 | 1.08 |
| *Risk—Liver or Kidney Problem* | 1.05 | 1.05 |
| *Mean VIF* | 2.18 | 1.53 |

Variance Inflation Factor

The variance inflation factor (VIF) reports the extent to which the standard error for each coefficient is increased due to the presence of multicollinearity. As the factor increases, so does the likelihood of committing a type II error. Generally, VIF statistics under 10.00 are considered acceptable [1]. As shown in S2 Table, the mean VIF of our primary model is sufficiently low, though the VIF for our key independent variables exceed traditional metrics. That said, this is due entirely to the inclusion of the quadratic term (i.e., *Science Trust—Apolitical* and *Science Trust—Apolitical^2^* are, definitionally, collinear) as evidenced by the VIF (=1.91) for an alternate model excluding the quadratic term. Given this, we can accept that our results are not severely impacted by multicollinearity.

Alternative Models

One potential concern is that our results may be dependent on control variable inclusion. Specifically, given the large number of control variables added to the model, one may be concerned that the results are driven by collider bias [2]. To test this, we first estimate a model that solely includes the key independent variables. We then estimate nine models that solely include the key independent variables, and the variables that correspond to each of the latent concepts identified in the main brief. As shown in S4 Table, our results are robust across all specifications. Also note that the Akaike Information Criterion (AIC) and Bayesian Information Criterion (BIC) values demonstrate that our primary model exhibits greater goodness of fit. Given that our primary model i) controls for a wider range of potential confounders, and ii) minimizes information loss, it is the preferred model.

**S4 Table. Regression Results for Alternate Multilevel Regression Models**

|  | (1) | (2) | (3) | (4) | (5) | (6) | (7) | (8) | (9) | (10) |
| --- | --- | --- | --- | --- | --- | --- | --- | --- | --- | --- |
|  |  |  |  |  |  |  |  |  |  |  |
| *Science Trust—Apolitical* | 0.60*** | 0.61*** | 0.53*** | 0.56*** | 0.54*** | 0.59*** | 0.55*** | 0.48*** | 0.47*** | 0.59*** |
|  | (0.03) | (0.03) | (0.03) | (0.03) | (0.03) | (0.03) | (0.03) | (0.03) | (0.02) | (0.03) |
| *Science Trust—Apolitical^2^* | -0.02*** | -0.02*** | -0.02*** | -0.02*** | -0.02*** | -0.02*** | -0.02*** | -0.02*** | -0.02*** | -0.02*** |
|  | (0.00) | (0.00) | (0.00) | (0.00) | (0.00) | (0.00) | (0.00) | (0.00) | (0.00) | (0.00) |
| *Science Trust—Betterment* |  | 0.04*** |  |  |  |  |  |  |  |  |
|  |  | (0.01) |  |  |  |  |  |  |  |  |
| *Science Trust—Community* |  | -0.01 |  |  |  |  |  |  |  |  |
|  |  | (0.01) |  |  |  |  |  |  |  |  |
| *Government Trust Index* |  |  | -0.26*** |  |  |  |  |  |  |  |
|  |  |  | (0.01) |  |  |  |  |  |  |  |
| *Media Trust* |  |  | 0.08*** |  |  |  |  |  |  |  |
|  |  |  | (0.01) |  |  |  |  |  |  |  |
| *Use of Science—Government* |  |  | -0.04*** |  |  |  |  |  |  |  |
|  |  |  | (0.01) |  |  |  |  |  |  |  |
| *Use of Science—Media* |  |  | 0.16*** |  |  |  |  |  |  |  |
|  |  |  | (0.01) |  |  |  |  |  |  |  |
| *Personal Impact—Infected* |  |  |  | -1.77*** |  |  |  |  |  |  |
|  |  |  |  | (0.13) |  |  |  |  |  |  |
| *Network Impact—Infected* |  |  |  | 0.26*** |  |  |  |  |  |  |
|  |  |  |  | (0.06) |  |  |  |  |  |  |
| *Personal Impact—Finances* |  |  |  |  | -0.07*** |  |  |  |  |  |
|  |  |  |  |  | (0.01) |  |  |  |  |  |
| *Personal Impact—Mental* |  |  |  |  | -0.10*** |  |  |  |  |  |
|  |  |  |  |  | (0.01) |  |  |  |  |  |
| *Network Impact—Finances* |  |  |  |  | -0.01 |  |  |  |  |  |
|  |  |  |  |  | (0.01) |  |  |  |  |  |
| *Network Impact—Mental* |  |  |  |  | -0.05*** |  |  |  |  |  |
|  |  |  |  |  | (0.01) |  |  |  |  |  |
| *Scientific Literacy* |  |  |  |  |  | 0.21* |  |  |  |  |
|  |  |  |  |  |  | (0.08) |  |  |  |  |
| *Individuals to Blame* |  |  |  |  |  |  | 0.24*** |  |  |  |
|  |  |  |  |  |  |  | (0.01) |  |  |  |
| *Individual Responsibility* |  |  |  |  |  |  | -0.23*** |  |  |  |
|  |  |  |  |  |  |  | (0.01) |  |  |  |
| *Dogmatism Index* |  |  |  |  |  |  |  | -0.22*** |  |  |
|  |  |  |  |  |  |  |  | (0.01) |  |  |
| *Race: Reference=White* |  |  |  |  |  |  |  |  |  |  |
| *Black* |  |  |  |  |  |  |  |  | -0.12 |  |
|  |  |  |  |  |  |  |  |  | (0.07) |  |
| *Hispanic* |  |  |  |  |  |  |  |  | 0.12 |  |
|  |  |  |  |  |  |  |  |  | (0.06) |  |
| *Other* |  |  |  |  |  |  |  |  | 0.54*** |  |
|  |  |  |  |  |  |  |  |  | (0.07) |  |
| *College Graduate* |  |  |  |  |  |  |  |  | -0.21*** |  |
|  |  |  |  |  |  |  |  |  | (0.04) |  |
| *Age* |  |  |  |  |  |  |  |  | 0.01*** |  |
|  |  |  |  |  |  |  |  |  | (0.00) |  |
| *Political Party: Reference=Democrat* |  |  |  |  |  |  |  |  |  |  |
| *Independent* |  |  |  |  |  |  |  |  | -0.14** |  |
|  |  |  |  |  |  |  |  |  | (0.05) |  |
| *Libertarian* |  |  |  |  |  |  |  |  | -0.68*** |  |
|  |  |  |  |  |  |  |  |  | (0.16) |  |
| *Other* |  |  |  |  |  |  |  |  | -0.03 |  |
|  |  |  |  |  |  |  |  |  | (0.10) |  |
| *Republican* |  |  |  |  |  |  |  |  | -0.55*** |  |
|  |  |  |  |  |  |  |  |  | (0.06) |  |
| *Trump Approval* |  |  |  |  |  |  |  |  | -1.40*** |  |
|  |  |  |  |  |  |  |  |  | (0.05) |  |
| *Risk—Pregnant* |  |  |  |  |  |  |  |  |  | -0.69*** |
|  |  |  |  |  |  |  |  |  |  | (0.16) |
| *Risk—Asthma* |  |  |  |  |  |  |  |  |  | 0.19** |
|  |  |  |  |  |  |  |  |  |  | (0.07) |
| *Risk—Lung Disease* |  |  |  |  |  |  |  |  |  | 0.17 |
|  |  |  |  |  |  |  |  |  |  | (0.15) |
| *Risk—Diabetes* |  |  |  |  |  |  |  |  |  | -0.18* |
|  |  |  |  |  |  |  |  |  |  | (0.07) |
| *Risk—Immune Disorder* |  |  |  |  |  |  |  |  |  | 0.63*** |
|  |  |  |  |  |  |  |  |  |  | (0.10) |
| *Risk—Obesity* |  |  |  |  |  |  |  |  |  | 0.18* |
|  |  |  |  |  |  |  |  |  |  | (0.07) |
| *Risk—Heart Problem* |  |  |  |  |  |  |  |  |  | 0.18* |
|  |  |  |  |  |  |  |  |  |  | (0.09) |
| *Risk—Liver or Kidney Problem* |  |  |  |  |  |  |  |  |  | -0.12 |
|  |  |  |  |  |  |  |  |  |  | (0.15) |
| *Constant* | 3.38*** | 3.19*** | 4.41*** | 3.50*** | 4.73*** | 3.26*** | 3.54*** | 4.89*** | 4.23*** | 3.35*** |
|  | (0.07) | (0.10) | (0.09) | (0.07) | (0.09) | (0.09) | (0.11) | (0.11) | (0.10) | (0.07) |
| *RE Variance: State* | 0.188*** | 0.188*** | 0.161*** | 0.189*** | 0.201*** | 0.190*** | 0.153*** | 0.201*** | 0.150*** | 0.197*** |
|  | (0.036) | (0.036) | (0.032) | (0.036) | (0.038) | (0.037) | (0.032) | (0.037) | (0.033) | (0.038) |
| *LR Test: χ^2^* | 45.09*** | 44.79*** | 36.66*** | 47.89*** | 49.41*** | 45.37*** | 31.63*** | 52.28*** | 24.70*** | 46.09*** |
| *AIC* | 54271.88 | 54196.83 | 53103.94 | 53996.30 | 53276.26 | 54267.81 | 52909.25 | 53935.79 | 52723.46 | 51903.00 |
| *BIC* | 54308.83 | 54248.55 | 53170.42 | 54048.02 | 53342.69 | 54312.15 | 52960.97 | 53980.13 | 52834.27 | 51998.50 |
| *Observations* | 11,967 | 11,952 | 11,932 | 11,949 | 11,866 | 11,967 | 11,943 | 11,964 | 11,941 | 11,459 |
| *Number of groups* | 51 | 51 | 51 | 51 | 51 | 51 | 51 | 51 | 51 | 51 |

Standard errors in parentheses: *** p<0.001, ** p<0.01, * p<0.05

**References**

1. O’brien RM. A caution regarding rules of thumb for variance inflation factors. Quality & quantity. 2007;41(5):673-690.
2. Greenland S, Pearl J, Robins JM. Causal diagrams for epidemiologic research. Epidemiology. 1999;37-48.
